# Control Plane Optimisation for an SDN-Based WBAN Framework to Support Healthcare Applications

**DOI:** 10.3390/s20154200

**Published:** 2020-07-28

**Authors:** Khalid Hasan, Khandakar Ahmed, Kamanashis Biswas, Md. Saiful Islam, A. S. M. Kayes, S. M. Riazul Islam

**Affiliations:** 1 School of Information and Communication Technology, Griffith University, 58 Parklands Dr, Southport, QLD 4222, Australia; 2 College of Engineering and Science, Victoria University, Ballarat Rd, Footscray, VIC 3011, Australia; khandakar.ahmed@vu.edu.au; 3 Faculty of Law and Business, Australian Catholic University, 8-20 Napier St, North Sydney, NSW 2060, Australia; kamanashis.biswas@acu.edu.au; 4 Department of Computer Science and Information Technology, La Trobe University, Melbourne, VIC 3086, Australia; a.kayes@latrobe.edu.au; 5 Department of Computer Science and Engineering, Sejong University, Seoul 05006, Korea; riaz@sejong.ac.kr

**Keywords:** Software-Defined Networking (SDN), Wireless Body Area Network (WBAN), SDN with WBAN (SDWBAN), Controller, SDN-enabled switches (SDESW), healthcare applications

## Abstract

Software-Defined Networking (SDN) offers an abstract view of the network and assists network operators to control the network traffic and the associated network resources more effectively. For the past few years, SDN has shown a lot of merits in diverse fields of applications, an important one being the Wireless Body Area Network (WBAN) for healthcare services. With the amalgamation of SDN with WBAN (SDWBAN), the patient monitoring and management system has gained much more flexibility and scalability compared to the conventional WBAN. However, the performance of the SDWBAN framework largely depends on the controller which is a core element of the control plane. The reason is that an optimal number of controllers assures the satisfactory level of performance and control of the network traffic originating from the underlying data plane devices. This paper proposes a mathematical model to determine the optimal number of controllers for the SDWBAN framework in healthcare applications. To achieve this goal, the proposed mathematical model adopts the convex optimization method and incorporates three critical SDWBAN factors in the design process: number of controllers, latency and number of SDN-enabled switches (SDESW). The proposed analytical model is validated by means of simulations in Castalia 3.2 and the outcomes indicate that the network achieves high level of Packet Delivery Ratio (PDR) and low latency for optimal number of controllers as derived in the mathematical model.

## 1. Introduction

The healthcare industry is advancing rapidly in providing remote healthcare services to patients with the assistance of information and communication technologies [1,2]. The WBAN is regarded as one of the pioneers in delivering remote healthcare services. The remote patient monitoring applications for various physiological data such as blood pressure, glucose level, calorie, temperature, heart rate, pulse rate measurement, etc. are now being feasible through WBAN [3,4,5]. On top of that, the overall management and operations of WBAN has become much more flexible and independent with the incorporation of SDN technology. Since the working principle of SDN offers programmable features in installing new applications irrespective of the devices designed by numerous vendors [6,7,8,9], the combination of SDNs and WBANs is considered to enhance the remote healthcare services to an outstanding level (see Table 1 for a list of abbreviations).

**Figure 1:**
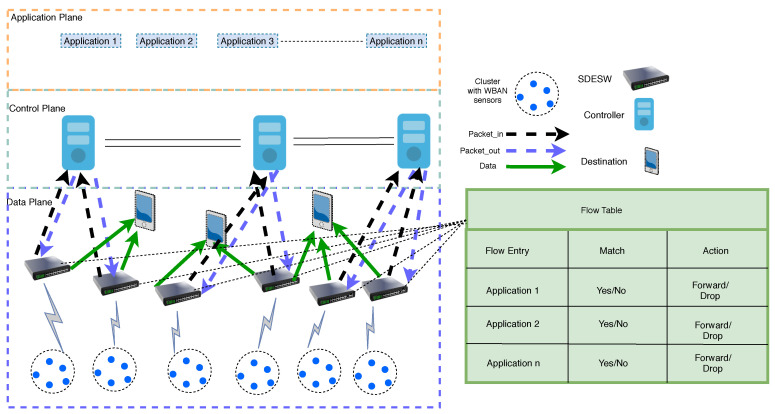
SDWBAN Framework.

The SDWBAN framework for patient monitoring applications simplifies the data packet forwarding functions from a source to a destination via SDN-enabled switches [10] as shown in Figure 1. The SDWBAN framework consists of three planes that reflect the basic principle of the SDN architecture. The data plane of the SDWBAN framework holds the WBAN sensors, SDESWs and gateways. The distributed controllers reside in the control plane which maintains communication with the underlying SDESWs in order to provide instructions for *packet_in* requests. On top of that, the healthcare authorities manage various sorts of applications through the application plane. In a nutshell, the working procedure of SDWBAN is as follows: the WBAN sensors at the data plane form clusters and connect with SDESWs. These sensors forward data to the connected SDESWs in order to reach out to the gateway. Upon receiving the data from body sensors, the SDESW checks for a match with the flow table and initiates a *packet_in* request to the controller in the case of mismatch. The controller then processes the request and sends a *packet_out* response to the SDESW with appropriate action instructions. The SDESW then forwards/drops the data packet based on the retrieved instructions from the controller. The detailed functionalities of the SDWBAN framework can be found in [10]. The SDWBAN becomes more complex as the number of applications and patients increase in the deployed area. A successful implementation and assurance of optimal performance in a complex SDWBAN largely depends on the appropriate design of the control plane. In the design of the SDN control plane, the number of controllers plays a crucial role in maintaining Quality of Service (QoS) and network performance. For wired SDN deployment, multiple controllers can reside in the control plane as the controllers have own dedicated physical resources to maintain inter-controller communication. However, in the case of wireless deployment, the controllers share the same in-band frequency and this could ultimately cause congestion while supporting inter-controller communication. As bandwidth is one of the scarce resources in the wireless medium, using multiple frequency bands for the control plane is very complex and hence, leads to further challenges such as interference, synchronization, etc. Therefore, choosing the optimal number of controllers to maintain the QoS in SDWBANs is imperative.

Even though a logically centralized controller can provide a global view of the network, a large-scale deployment of a SDWBAN has several limitations in regard to performance and scalability [11]. This is obvious that having a multiple number of controllers in the control plane, can relax the bottleneck of excessive load on a single controller. However, in order to maintain an abstract view of the network, the controllers need regular state synchronization [12]. This synchronization enables the controllers to support the underlying SDESWs with their queries for unknown applications or data forwarding instructions. Another issue is that the formation of the clusters is not fixed in a SDWBAN. Similarly, the number of supporting application groups under the SDEWSs could also vary from scenario to scenario. As such, when the cluster size is bigger, the probability of receiving more *packet_in* requests gets higher. Hence, it is vital to have a sufficient number of controllers in the control plane so that the originating *packet_in* requests from the SDESWs respond within an acceptable time frame. Consequently, an optimum number of controllers is required so that the SDWBAN supported healthcare applications ensure reliability and the timely delivery of physiological data. On the other hand, the redundant use of controllers in the design of the control plane is an unnecessary waste of resources and thus, adds redundant complexity.

To address the issues and limitations discussed above, it is vital to design an optimal control plane for a specific network. In the case of deploying i.e., SDWBAN, the design of the control plane is significant to ensure acceptable network performance in terms of PDR and latency. The optimal design of the control plane would be able to respond to the *packet_in* requests originating from all SDESWs. In this paper, a mathematical model is developed based on three influential factors of SDWBANs i.e., number of controllers, latency and number of SDESWs. The proposed mathematical model institutes a relationship between the number of controllers, SDESWs and the body sensors. The latency factor consists of important parameters related to the communication between the controller and the SDESW. The contributions of this paper are twofold:

- Development of a mathematical model that determines the optimal number of controllers for an SDWBAN framework. In addition, the mathematical model also establishes a relationship between the number of controllers and SDESWs.
- Implementation and validation of the proposed mathematical model through the Castalia simulator.

The rest of the paper is organized as follows: Section 2 presents the related works. Influential factors related to the optimization of control plane are presented in Section 3. Section 4 describes the mathematical model developed for SDWBAN framework optimization. Section 5 presents the analytical outputs of the mathematical model as well as experimental results. Finally, Section 6 concludes the paper with future research directions.

## 2. Related Works

The design of the control plane occupies a major role in maintaining the optimum level performance and QoS in an SDN deployed network. Several works pertaining to the design of control planes for SDN-based deployment on a large-scale have been published. However, the development of an optimization model for the SDWBAN framework has remained unexplored. This section provides a brief overview of some of the significant control plane design works that led to the development of the proposed optimization model.

One of the first works related to the design of the control plane of SDN was done by Heller et al. [13]. This work was motivated by three types of SDN users, namely network operators, controller application writers, and network management software writers. Being motivated by these instances, the authors analysed the controller placement problem (CPP) which addresses questions related to the number of controllers to use for a given topology and the placement of the controllers in the given topology. The authors defined a primary metric for the placement of the controllers and evaluated the average propagation latency of the control plane based on Euclidian distance. They also offered a solution based on the *k*-median and *k*-center algorithm. However, the solutions were stringently based on the average and worst case delay between the network elements. In the proposed method, decision to find the placement of the controllers was based on a brute force approach with the evaluation of possible locations. However, the approach did not consider traffic load variation and the dynamic adaptation with the number of controllers and position. More comprehensive work related to the CPP solution can be found in [14,15,16].

The flow setup delay in setting up paths between the controller and the switch is considered in [17] for wired SDNs. The functions of the proposed model are based on the activation and de-activation of the links throughout the network whenever required. The paper considers link cost, equipment cost, the capability of the controllers, path setup delay and traffic patterns to determine the optimal number and location of the controllers. The prime focus of the work is to minimize the financial cost involved in the installation and removal of network elements. On the other hand, our work focuses on finding the optimal number of controllers for SDWBAN. In addition, we provided simulation-based results that validate our proposed analytical outputs with the simulation-based output ones for a certain delay boundary. This clearly defines that our contribution conveys an added value in the context of the optimization of control plane in SDWBAN.

Hock et al. [18] designed a trade-off mechanism among various placement of controllers that comprises different control latency and controller overheads. The authors used a mechanism called Pareto-based Optimal Controller (POCO) placement that enables a decision to be made by exploring the solution space and performing various analysis through a GUI. The weakness of this approach is that the controllers require a lot of link state information which affects inter-controller latency.

A network partitioned-based controller placement strategy is proposed in [19] that employs a *k*-center algorithm in order to assist load balancing and network stability. In this work, the authors considered heterogeneous data plane traffic for optimization. In contrast, Jimenez et al. [20] considered homogeneous type of traffic for optimal controller placement. However, both of these works are restricted to the initial observation of constant traffic load and neglect the issue of dynamic traffic load adaptation.

An optimization model for the deployment of controllers and sinks for wireless sensor networks (WSN) is proposed in [21]. The prime focus of the proposed model is to determine the optimal location of the controllers and sinks to maintain reliability and performance in a delay sensitive Internet of Things (IoT) system. Similarly, Wei et al. in [22] proposed a two-level hierarchy control framework for SDN-based IoT networks to relax the bottleneck with the growing number of IoT devices. The authors addressed the issue of a controller placement strategy based on the priority of the nodes and use a binary particle swarm optimization (BPSO) algorithm to optimize the control performance of the SDN-based IoT network. In addition, Kushan et al. in [23] discussed the optimization of controller placement for a hierarchical distributed software-defined vehicular network (SDVN). The prime focus of the work is to find the optimal placement of the controllers to reduce operational latency by locally distributing the top layer of the controllers while the bottom layer of the controllers is placed near the road side unit (RSU). Nevertheless, the studies in [21,22,23] do not consider the optimal number of controllers in their proposed models and scenarios.

An effective controller placement strategy was designed by Bagga et al. [24] that withstand the quality of services, although drops the network cost for an SDN-based 5G system. Another study focuses on the distributed controller placement architecture for wireless sensor network that fortifies the scalability [25]. Even though, these studies can yields decent output in terms of control cost or reliability, but overlooked the variance in importance of diverse network nodes. Gorkemli et al. [26] propose an approach for designing a programmable and distributed control plane architecture consisting of multiple controllers, out-of-band and in-band control channels that can be managed dynamically at scale. The authors introduced a “control flow table” where the rules can be embedded of a switch to manage in-band control flows and off-load the congested controllers and in-band control channels. The proposed architecture has been experimented over multiple topologies to demonstrate its scalability and performance superiority.

The aforementioned works are mostly related to the optimal location of controllers for the SDN-based network. The aim of optimization differs based on the nature of deployment and the requirements. Therefore, the problems and solutions related to the optimal control plane design have been discussed from various angles in the literature. However, our work is represented differently compared to the aforementioned studies. Since the timely delivery of data is of paramount significance in WBAN, the total flow resolution time sets a boundary limit for healthcare applications. Therefore, several flow resolution-related parameters are taken into account in developing a mathematical model to find the optimal number of controllers for the SDWBAN framework. To the best of our knowledge, the optimization model presented in this paper is the first attempt to find the optimal number of controllers in an SDWBAN framework.

## 3. Influential Factors of Optimization

There are several factors that play vital roles in the design of a control plane for SDWBAN implementation. These important factors are described as follows.

- **Number of Controllers:** The number of controllers plays a crucial role in the design and implementation of an optimal SDWBAN framework. The installation of a large number of controllers increases the complexity and cost of network management. The processing capacity of commercially available controllers typically varies, for instance, the processing speed of an industrial controller is usually very high and capable of maintaining millions of client devices simultaneously [27,28]. In such a case, the use of industrial controllers for SDWBAN deployment in an elderly home would be redundant. Since responding to the *packet_in* request initiated by the SDESW mostly depends on its processing speed, it is desirable to have an optimum number of controllers in SDWBAN deployment. The objective is to ensure a well-maintained communication between the controller and the SDESW and thus, patient monitoring activities are not compromised at all.
- **Latency:** Latency is related to several factors such as flow request processing time, propagation delay, service rate etc. In SDWBAN deployment, if an inadequate number of controllers is deployed, the controllers might undergo a considerable amount of delay in route setup. Consequently, sending out the data forwarding instructions to the SDESW will be affected in terms of flow requesting resolving time. In addition, there could be an increased amount of traffic load in the control channel communication between SDESW and the controller as new applications are introduced in the system. Ultimately, the latency incurred by this will affect the overall network performance in terms of packet delivery which will hamper patient monitoring activities.
- **Geographical Location:** The geographical location of controllers and SDESWs is another important factor in designing the control plane. The issue of line of sight (LOS) and non-line of sight (NLOS) exists in wireless network deployment between the transmitter and the receiver. The NLOS scenario could be due to the fact that the patients in SDWBAN are at liberty to roam around, and in addition, the placement of a particular application sensor might block the direct propagation from body sensors to the SDESW. Similarly, other objects such as the walls of buildings, deflections at sharp edges, and multipath propagation may affect the signal strength in an SDWBAN environment. Considering these factors, it is important to design a system that maintains a standard receiver sensitivity level. Furthermore, an arbitrary placement of network elements (controllers, SDESWs, gateway) would cause additional propagation delay between the network elements and ultimately increase overall latency.
- **Traffic Load distribution:** The number of SDESWs residing under a controller is an important issue in determining traffic load. If the number of SDESWs is high under a controller, the probability of receiving a *packet_in* request also increases. Consequently, the number of *packet_out* responses to the SDESW also increases. As a result, traffic load increases between the communication channel of the controller and the SDESW. Considering the processing capacity of a particular type of controller, SDN controllers can be programmed in such a way that the excess load can be distributed to the neighboring controllers.
- **Flow Setup Time:** The flow setup time is the amount of time a SDESW needs to send a query to the controller to install data forwarding rules in its flow table. If the number of flow requests originating from the underlying SDESW is larger than the processing capacity of the controller, the average flow setup time can increase significantly which will degrade the service performance [29,30].
- **Statistics Collection Time and Synchronization Cost:** In the case of multiple controllers residing in the control plane, inter-controller communication takes place to maintain an abstract view of the network and this assists the controllers to provide data forwarding decisions to the switches [11]. The controllers deployed in the network can be inter-connected so that upon failure of one controller, the next available controller can serve the orphaned SDESWs. Moreover, to maintain a consistent view of the network, it is important to keep the synchronization between the controllers [12]. The controllers can have an overall view of the network by collecting various statistics such as port information, flows, flow table level etc. from the switches. This requires several messages to be exchanged between the controllers and switches. Consequently, a trade-off is necessary between the flow setup time and statistics collection time in order to avoid delay in flow resolution.
- **Number of SDESW:** The number of SDESWs in the deployed area could be a crucial point and may create a bottleneck in the network. With a higher number of SDESWs residing in the network, a higher number of flow requests are initiated to the controllers upon realizing an unknown flow. This will ultimately affect the PDR and latency of the network.

In a nutshell, in SDN deployment as well as in SDWBAN, various factors influence the performance of the network. The aim of the optimization of any network is to achieve the optimum level of performance that satisfies the purpose of the deployed application. When the network becomes increasingly complex, more factors will play critical roles in the performance of the network. To make the system robust and more secure, various influential factors and functionalities mentioned above can be incorporated in SDN deployment. However, not all the above-mentioned factors might be crucial for wireless deployment i.e., in SDWBAN deployment. From the perspective of healthcare applications, the issues relating to geographical location can be handled by careful topology design whereas a dynamic load balancing algorithm can effectively distribute traffic load among the controllers [31]. On the other hand, flow setup time and statistic collection time and synchronization cost are directly associated with the number of SDESWs and SDN controllers respectively. Since patient monitoring in SDWBAN should maintain a strict delay boundary, out of all the influential factors related to the optimization of the control plane, we restrict our optimization constraints within three crucial factors: number of controller, latency and the number of SDESWs. The rest of the factors are not elaborated in this work as these factors are not within the scope of this work. The interested readers can look into the given references for more details.

## 4. Development of Optimization Model

This section describes the optimization aspects in the SDWBAN framework and proposes a mathematical model to support the SDWBAN scenario.

### 4.1. Optimization in SDWBAN Scenario

**Figure 2:**
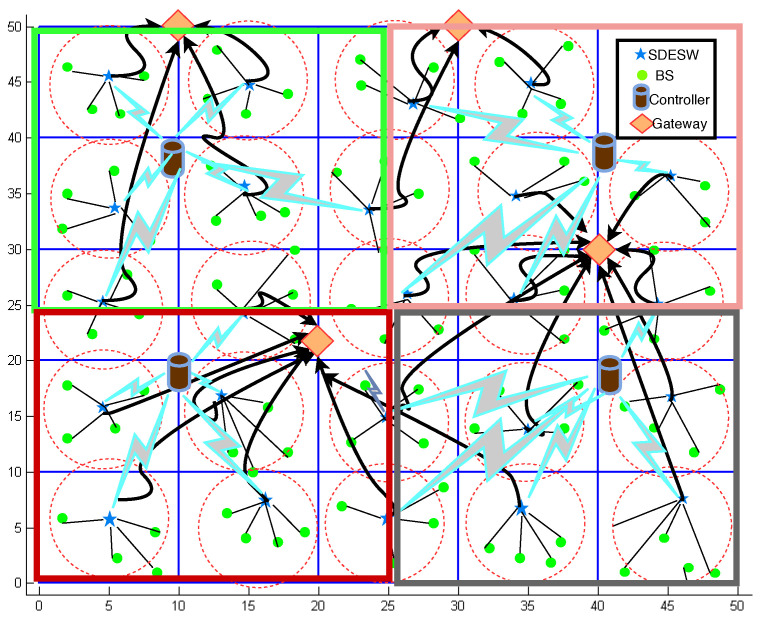
SDWBAN Implementation Scenario (adapted from our previous work Hasan et al. [32]).

The initial work of SDWBAN [32] concerns the validation of the proposed framework and the impact of the application classification algorithm. The implementation scenario of the initial work is depicted in Figure 2. In this case, the simulation area of 75 ×75 m${}^{2}$ consists of four intuitively chosen controllers and randomly deployed body sensors. The SDESWs are deployed in a static fashion. The body sensors form clusters in various sectors and associate with a SDESW. The SDESWs maintain communication with the controllers for data forwarding instructions. The aim of this design is to observe the performance of the network in terms of PDR and latency for several applications’ data originating from the data plane. The primary implementation of this particular scenario performs satisfactorily with the specific number of applications, SDESWs and controllers. However, in the case of the scaling up or down of heterogeneous applications, the performance might degrade as well. Furthermore, since the WBAN applications could include emergency data, responding to such events should be given the utmost priority to avoid undesirable circumstances. Therefore, an optimal design for the control plane is imperative so that the residing number of controllers are capable of supporting the data plane functionalities.

### 4.2. Proposed Mathematical Model

According to SDN principle, controllers are responsible for installing flow commands in a switch to route the data packets to appropriate destinations. As such, when an SDESW makes a query about an unknown traffic, the controller processes the query and replies back to the SDESW with appropriate flow commands. Thus, the time to process a flow is a crucial factor in identifying the optimal point for the implementation of the SDWBAN framework.

Let us assume that *T* is the total flow resolution time of a controller to resolve an incoming flow request and to respond to the request to a SDESW. We get,

(1) $$T=T_{FR}+T_{q}+T_{proc}+T_{prop1}+T_{prop2}+T_{RD}$$

where $T_{FR}$, $T_{q}$, $T_{proc}$, $T_{prop1}$, $T_{prop2}$ and $T_{RD}$ represent flow request delay, queuing delay, processing delay, propagation delay from SDESW to controller, propagation delay from controller to SDESW and relaying delay, respectively. These notations are summarised in Table 2. It should be noted that these time parameters in Equation (1) (and the parameters in the subsequent equations of our model) represent expected values of random variables.

The Flow request delay ($T_{FR}$) is associated with a SDESW. This is the time it takes to realize there is a new flow which is not in the flow table and to send the query to the controller by sending a *packet_in* request. $T_{FR}$ depends on the matching probability in the flow table and the speed of the flow look-up process.

The Queuing delay ($T_{q}$) is associated with the controller. This is the time experienced by each packet while waiting in the queue of a controller. Let us assume that each SDESW and controller maintains a single finite queue, which can be modelled by using the M/M/1/K queuing model. The packet arrival rate from a SDESW to a controller due to the unmatched flow is assumed to be a Poisson distribution. Let us assume, the mean packet arrival rate from a SDESW is λ, service rate at the controller is α, *K* is the maximum queue size at the controller, and use factor ρ=λ/α.

Therefore, according to [23], queuing delay $T_{q}$ can be represented by the following equation:

(2) $$T_{q}^{{}^{\prime }}=\frac{\rho [1-\rho^{K}-K\cdot \rho^{K-1}\cdot \left(1-\rho \right)]}{\alpha \left(1-\rho \right)\left(1-\rho^{K+1}\right)}$$

Again, $\bar{\lambda }=\lambda \cdot \left(1-P_{K}\right)$, where $P_{K}$ is the probability of exactly K packet in the queuing system and $\bar{\lambda }$ is the average packet arrival rate over the long run. Therefore, from Equation (2),

(3) $$T_{q}^{{}^{\prime }}=\frac{\lambda [1-\rho^{K}-K\cdot \rho^{K-1}\cdot \left(1-\rho \right)]}{\alpha \cdot \alpha \left(1-\rho \right)\left(1-\rho^{K+1}\right)}$$

and,

(4) $$T_{q}^{{}^{\prime }}=\frac{\bar{\lambda }\left[1-\rho^{K}-K\cdot \rho^{K-1}\cdot \left(1-\rho \right)\right]}{\left(1-P_{K}\right)\alpha \cdot \alpha \left(1-\rho \right)\left(1-\rho^{K+1}\right)}$$

Assuming $P_{K}\to 0$, $\bar{\lambda }=\lambda$ and the packet arrival rate from the SDESWs will never exceed the size of the queue. Therefore, Equation (4) can be considered to be,

(5) $$T_{q}^{{}^{\prime }}=\frac{\lambda [1-\rho^{K}-K\cdot \rho^{K-1}\cdot \left(1-\rho \right)]}{\alpha \cdot \alpha \left(1-\rho \right)\left(1-\rho^{K+1}\right)}$$

Assuming the similar packet arrival rate from θ number of SDESWs in the deployed area, queuing delay $T_{q}$ can be expressed as,

(6) $$T_{q}=\theta \cdot T_{q}^{{}^{\prime }}$$

The Processing delay ($T_{proc}$), is associated with the controller and depends heavily on the processor’s speed. This is the time taken by a controller to process a flow-request (*packet_in*) and reply to the SDESW with flow commands. The processing delay can be expressed as the inverse of service rate α. Therefore,

(7) $$T_{proc}=\alpha^{-1}$$

Propagation delay ($T_{prop1},T_{prop2}$) occurs when a flow request is sent from a SDESW to the controller and from the controller to a SDESW with an appropriate flow command (packet_out). By considering,

(8) $$T_{prop1}≅T_{prop2}≅T_{prop}$$

where $T_{prop}$ is the total propagation delay and from Equations (1), (6) and (8),

(9) $$T=T_{FR}+\theta \cdot T_{q}^{{}^{\prime }}+T_{proc}+2\cdot T_{prop}+T_{RD}$$

Relaying delay ($T_{RD}$) is associated with the SDESW. Let us assume, we have multi-hop communication between the SDESW and the controller. The flow request originating at the SDESW reaches out to the controller through multi-hop relay. Therefore, in each hop, the SDESW stores the request packet and forwards it to the next SDESW and so on until it reaches the controller. Let’s say there are *n* number of hops between a SDESW and a controller and $T_{SF}$ is storing and forwarding delay which is a two-way computation associated with *packet_in* and *packet_out* responses. Therefore,

(10) $$T_{RD}=2\cdot n\cdot T_{SF}$$

Since we are considering *n* number of hops between a SDESW and a controller, the propagation delay needs to be associated with the number of hops as well. The propagation delay, $T_{prop}$ can be written as,

(11) $$T_{prop}=n\cdot T_{hop}$$

where, $T_{hop}$ is the propagation delay in one hop between a SDESW and a controller. Therefore, from Equations (9)–(11),

(12) $$T=T_{FR}+\theta \cdot T_{q}^{{}^{\prime }}+T_{proc}+2\cdot \left(T_{hop}+T_{SF}\right)\cdot n$$

**Figure 3:**
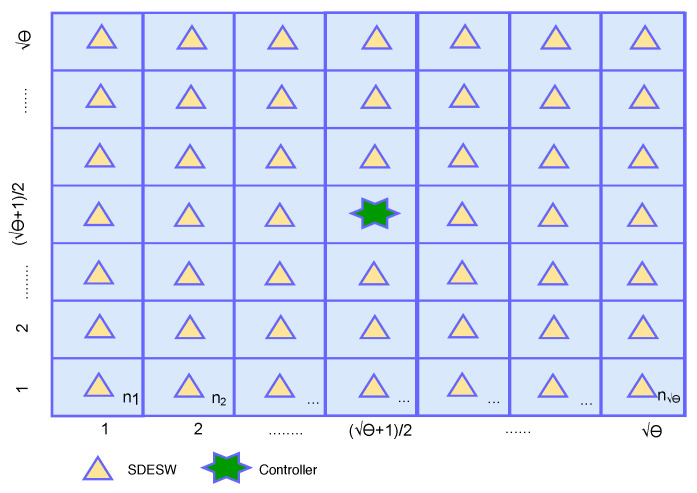
Hops in Building Grid.

**Lemma** **1.** *Assume there are θ number of SDESWs uniformly distributed in a $(\sqrt{\theta }$ X $\sqrt{\theta })$ building grid with one SDESW in each cell as depicted in Figure 3. A controller is deployed in the middle of the grid and all θ SDESWs are assigned to this controller. Then the average number of hops from a SDESW to controller is $\sqrt{\theta }/2$. The proof is given in the Appendix A.*

Then Equation (12) can be rewritten as,

(13) $$T=T_{FR}+\theta \cdot T_{q}^{{}^{\prime }}+T_{proc}+2\cdot \left(T_{hop}+T_{SF}\right)\cdot \frac{\sqrt{\theta }}{2}$$

Hence,

(14) $$\theta \cdot T_{q}^{{}^{\prime }}+\sqrt{\theta }\left(T_{hop}+T_{SF}\right)+T_{FR}+T_{proc}-T=0$$

(15) $${\left(\sqrt{\theta }\right)}^{2}\cdot T_{q}^{{}^{\prime }}+\sqrt{\theta }\left(T_{hop}+T_{SF}\right)+T_{FR}+T_{proc}-T=0$$

Let us assume that, $a=T_{q}^{{}^{\prime }}$, $b=(T_{hop}+T_{SF})$, and $c=T_{FR}+T_{proc}-T$. Therefore, from equation 11, it can be written,

(16) $$a{\left(\sqrt{\theta }\right)}^{2}+b\sqrt{\theta }+c=0$$

The solution of the quadratic Equation (12) is,

(17) $$\sqrt{\theta }=\left[\frac{-b\pm \sqrt{b^{2}-4ac}}{2a}\right]$$

The solution to Equation (17) will identify the optimal number of SDESWs (θ) under a single controller in a network. By substituting the coefficient values in Equation (17), we find the number of SDESWs per controller,

(18) $$\sqrt{\theta }=\left[\frac{-\left(T_{hop}+T_{SF}\right)\pm \sqrt{{\left(T_{hop}+T_{SF}\right)}^{2}-4T_{q}^{{}^{\prime }}\left(T_{FR}+T_{proc}-T\right)}}{2T_{q}^{{}^{\prime }}}\right]$$

The solution to Equation (18) will provide two different values of $\sqrt{\theta }$ that satisfy the quadratic equation. According to our assumption, the numerical coefficient “*c*” contains the parameter “*T*”, which is our total flow resolution time constraint, therefore we find “*c*” for a range of “*T*” and thus find the $\sqrt{\theta }$.

**Figure 4:**
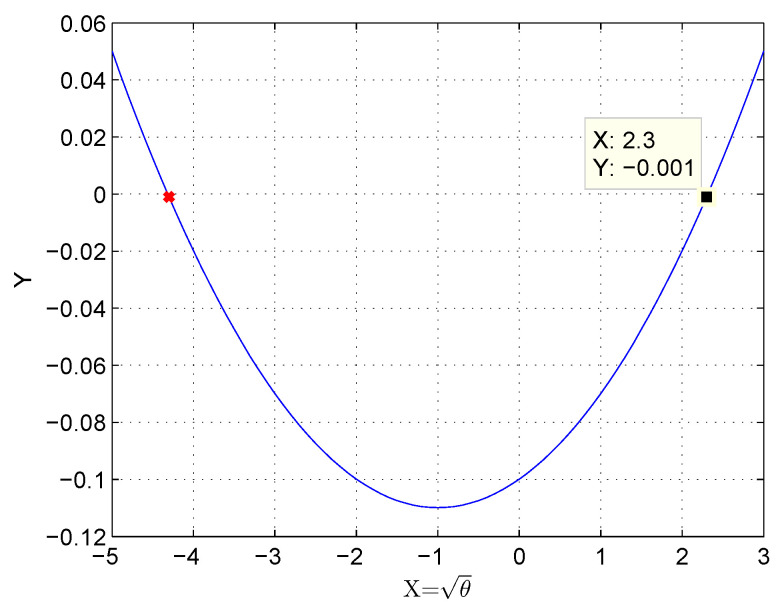
Intercept of quadratic equation (for T=110 ms and $y=a{\left(\sqrt{\theta }\right)}^{2}+b\sqrt{\theta }+c$).

It can be seen from Table 3 that for a fixed delay requirement, we get two different values for $\sqrt{\theta }$ that satisfy the derived quadratic equation. Based on our analysis, for a range of flow resolution time, $\sqrt{\theta }$ we get non-integer value. The possibility of obtaining $\sqrt{\theta }$ as integer depends on the related parameters considered in the quadratic equation (Equation (18)). To illustrate, for the flow resolution time of 110 ms, the intercept of the quadratic equation is given in Figure 4.

Since it is necessary to find the optimal number of SDESWs per controller, the negative part of the solution is avoided and thus, the valid solution of the quadratic equation is $\sqrt{\theta }$, 2.3155.

Let us assume that the packet arrival rate at the SDESW is δ, *s* is the number of WBAN sensors under each SDESW, and ϵ is the packet generation rate at each sensor, Therefore,

(19) δ=s·ϵ

If a SDESW, SW can accommodate κ number of packets per second and the total number of body sensors in a WBAN is *S*, the outcome of the following convex optimization problem derives the optimal number of SDESW required in the network [33].

(20) $$SW_{opt}=\frac{S}{s},\mathrm{subject}\;\mathrm{to}\;s\leq \frac{\kappa }{\epsilon }$$

The optimal number of controllers $C_{opt}$ for a certain delay boundary is,

(21) $$C_{opt}=\left[\frac{SW_{opt}}{\theta }\right]$$

here, $C_{opt}$ is an integer.

It should be noted that we have avoided the stochastic process of incoming flow request in our model for the sake of simplicity. For instance, queuing delay could be further elaborated by taking the sum of the mean packet arrival rates of the individual Poisson process at the SDESW; however, for the sake of simplicity, we have avoided the complex terms here.

## 5. Results and Analysis

This section details the results in two sub-sections. Firstly, the result of the optimization is discussed and secondly, the performance analysis of a SDWBAN implementation is presented in relation to the optimized number of controllers.

### 5.1. Analytical Output

**Figure 5:**
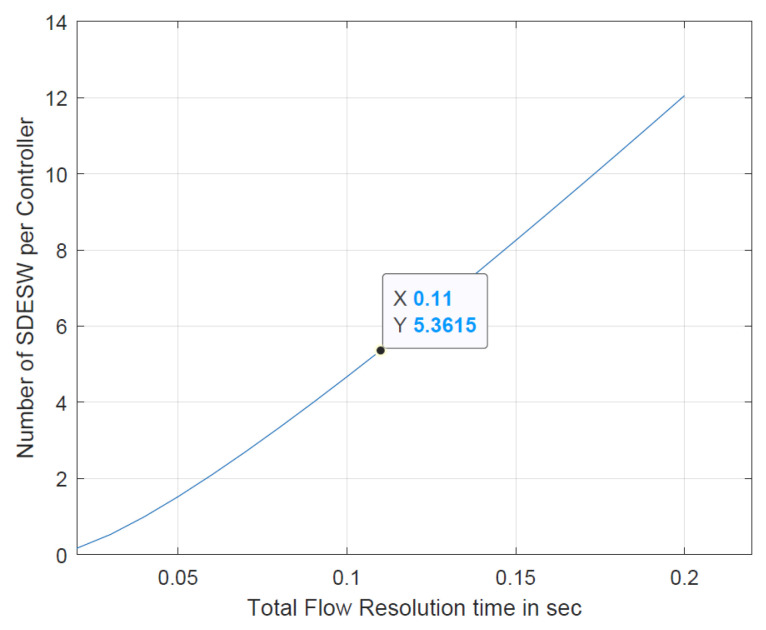
Number of SDESW per Controller.

Based on the derived mathematical model in Equation (18), the relationship between the number of SDESWs per controller and the total flow resolution time is presented in Figure 5. The graph shows that for a total resolution time of 110 ms, the number of SDESWs under a controller is 5.3615 ≈6. Ultimately, the number of SDESWs per controller for a total resolution time of 110 ms leads to finding the optimal number of controllers for the implementation of SDWBAN. Therefore, based on the primary work of SDWBAN implementation [32], for a total number of 100 body sensor nodes and 4 nodes under one SDESW, the optimal number of controllers is 5. Similarly, based on the optimization model, the required number of controllers for a range of body sensors is provided in Table 4. The list of parameters assumed in the mathematical model is given in Table 5.

Some significant observations can be noted from the obtained mathematical model. The observations are listed as follows:

- The optimal number of controllers required in an implementation scenario largely depends on the processing capacity of the controller. For instance, it is assumed that the service of the controller is 100 pkt/sec, which is based on simulation output. It is found from the simulation that the processing rate of a light-weight controller is 100 pkt/s. However, industrially available controllers have higher processing capacity [35]. The controllers with higher processing capacity or service will result in a faster response to the incoming *packet_in* request from a SDESW. The consequence will be low queuing delay due to the variable packet arrival rate from the underlying body sensors.
- The flow request delay is another important factor since it depends on the matching probability in the flow table and the speed of the look-up process.
- The packet arrival rate from the heterogeneous sensors affects the performance of the SDESW and the controller. Since the heterogeneous nature of WBAN sensors generates data packets at various intervals, this could imbalance the traffic load in the communication channel between the SDESW and the controller.
- The number of controllers and the number of SDESWs affect the overall performance. If there are fewer controllers in the deployed area, this limited number of controllers might have to cater for all the SDESWs under the assigned controller. In such a case, packet forwarding might route in a multi-hop fashion to the controller if the distance between the SDESW and the controller is out of transmission range. Consequently, it degrades the performance in terms of success rate and latency. In practice, various WBAN applications require to maintain various delay constraints. From the literature, based on the general WBAN guideline, a strict delay of 110 ms–120 ms is required to maintain.

### 5.2. Simulation Output

In this part of analysis, the simulation is conducted in Castalia 3.2 [36] on Ubuntu 16.04.4 platform. Each experiment is conducted for 100 iterations and the average value has been computed for the performance metrics of the given SDWBAN scenario. In the initial work [32], the experimental setup has used a total number of four controllers. However, in this case, the number of controllers is varied while the number of SDESWs remains fixed. The simulation is run for several groups of healthcare applications where the number of applications is incremented gradually. The number of applications per group is listed in Table 6. We have considered strict delay boundary of 110 ms for different applications, which is crucial for WBAN. It is very important to maintain this strict delay boundary in order to avoid undesirable circumstances in patient monitoring.

It should be noted that the simulation area and the other related parameters in this work are kept similar to the initial work. These parameters are summarised in Table 7. A rectangular area of 75 × 75 m${}^{2}$ is considered in the simulation and the body sensors (BSs) are deployed randomly. The BSs finds the received signal strength indicator (RSSI) from the corresponding SDESW and calculates the distance of the corresponding SDESW using the time of flight (TOF) principle. Then, the BS-SDESW association is completed on the basis of rank number calculated from RSSI and TOF. Thus, clusters form in different sector. For each group of applications, the simulation is conducted by varying the number of controllers from 1 to 10. Since the aim of this research is to find the optimal number of controllers for an SDWBAN framework, the simulation is run for a different number of controllers to obtain the average PDR and latency for each application group.

**Figure 6:**
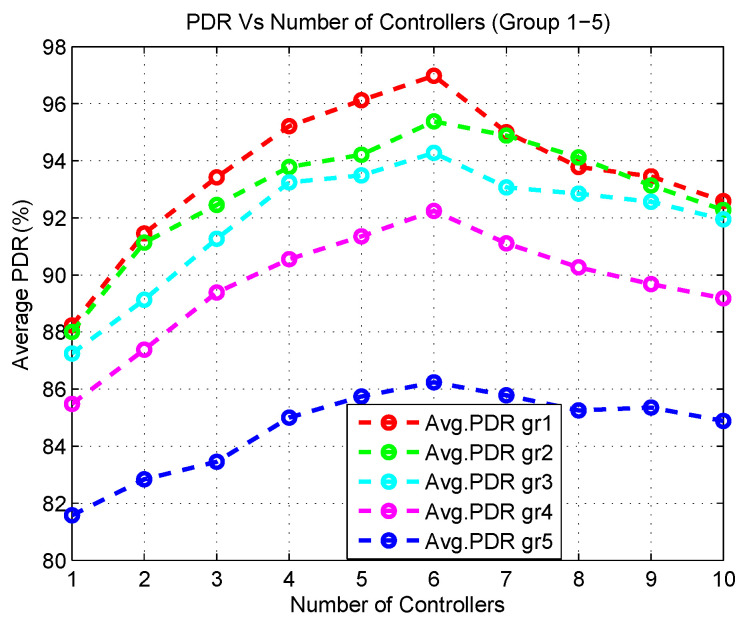
Average PDR with varying number of controllers.

In the first stage, the average PDR (PDR is defined as the ratio of the number of packets resolved to the number of packets transmitted) with a varying number of controllers is depicted in Figure 6. It can be seen that when the number of controllers is low, the PDR for all groups (group 1–5) is also low and when the number of controllers starts to increase, the PDR increases as well. However, at some point, the PDR starts to decrease even when the number of controllers increases. For instance, when the number of controllers ranges from 1 to 3, the average PDR increases. When the number of controllers ranges from 4 to 6, the average PDR still increases. However, after the 6th number of controllers, the average PDR decreases. The reasons of this are as follows:

- When the number of controllers is low (1, 2, or 3), less controllers are used to cater for a lot of flow resolve requests from a various number of application groups. This creates a bottleneck to respond to the incoming *packet_in* requests coming from the SDESWs. Furthermore, a lot of multi-hop communication takes place since the destination controller is beyond the transmission range of SDESWs. This results in a delay in accessing the channel. In addition, the traffic load increases between the communication channel of SDESWs and the controllers. Thus, the PDR of all application groups decreases.
- When the controller number is between 4 to 6, the average PDR shows little variation in the output which actually shows the optimal range of controllers that are required for the SDWBAN framework.
- When the number of controllers is between 7 to 10, the PDR decreases due to the fact that a lot of controllers reside nearby and use in-band frequency. This also causes congestion and interference with the neighbouring nodes in the communication channel.

**Figure 7:**
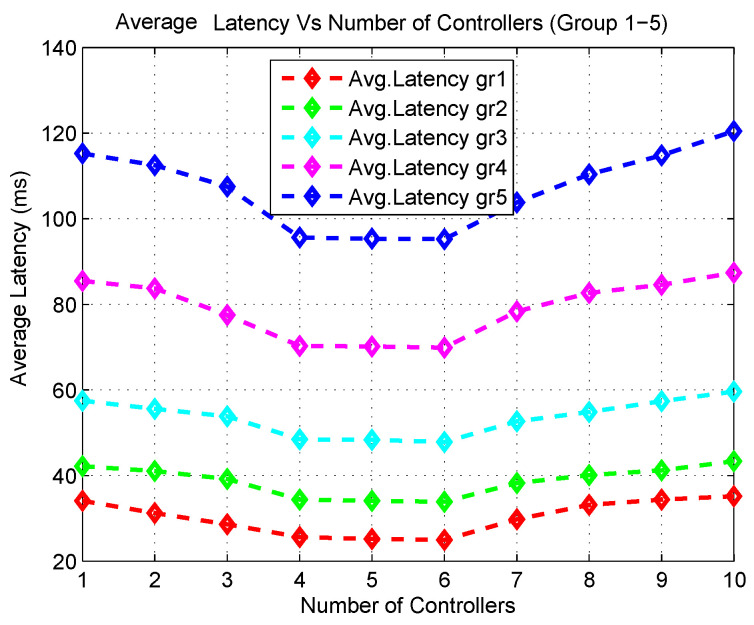
Average Latency with varying number of controllers.

The simulation output is further observed by analyzing the average latency for a different number of application groups. The average latency depicted in Figure 7 shows that initially with a low number of controllers, all application groups experience high latency. The average latency stays more or less stable when the number of controllers is between 4 and 6 but with an increase in the number of controllers, latency increases. This phenomenon coincides with the facts of average PDR output.

From the simulation outcomes of average PDR and latency, it can be seen that the best performance is received from the network when the number of controllers is set to 6. Although the PDR gain is high when using 6 controllers, no significant improvement in the latency is noticed in the outcomes. In fact, the average latency remains almost the same for 4, 5 and 6 controllers. These outcomes indicate that the network would be able to achieve its peak performance when the number of controllers is set to 5. Since latency is one of the key elements in the proposed mathematical model, the experimental results validate the analytical outcome derived from the mathematical model for 100 body sensors.

**Figure 8:**
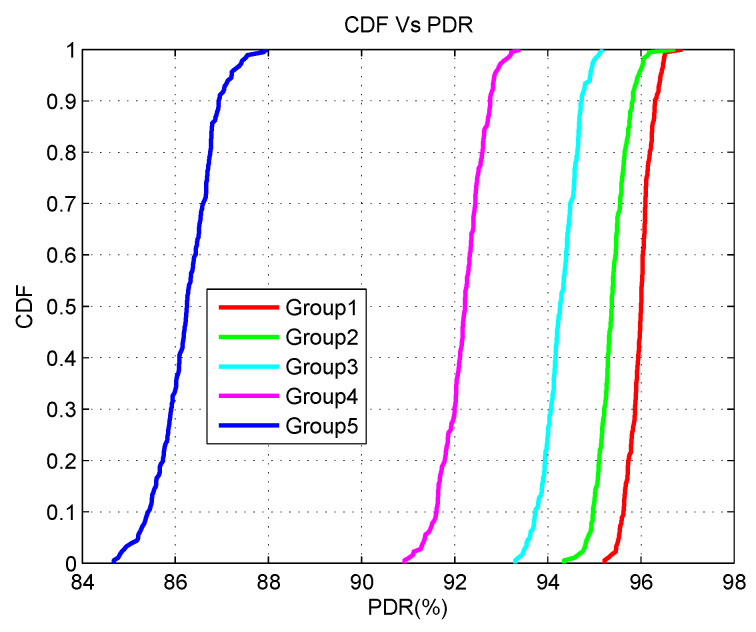
CDF Vs PDR.

The experimental output is further elaborated on for the cumulative distribution function (CDF) of PDR and latency for the optimal five controllers. Figure 8 presents the output for a different group of applications in terms of PDR. According to Figure 8, group1 has the highest PDR while group5 exhibits the lowest PDR. A similar type of variation can be noticed in the PDR of the other groups (2–4). The reason for this is due to the increased number of applications. As the number of applications increases, the SDESW initiates more *packet_in* requests for every unknown flow. Therefore, the traffic load between the control channel of SDESWs and the controller increases. Thus, more data packets are dropped due to congestion. As can be observed from the group1 graph, at the 90th percentile point, the PDR of group1 is 96.38% whereas the PDR of group5 is 86.78%. This performance demonstrates that any packet of group1 at the 90th percentile has a 96.38% probability of being delivered successfully and a 3.62% probability of being dropped. Similarly, any packet of group5 at 90th percentile point has a 86.78% probability of being delivered successfully whereas it has a 13.22% probability of being dropped.

**Figure 9:**
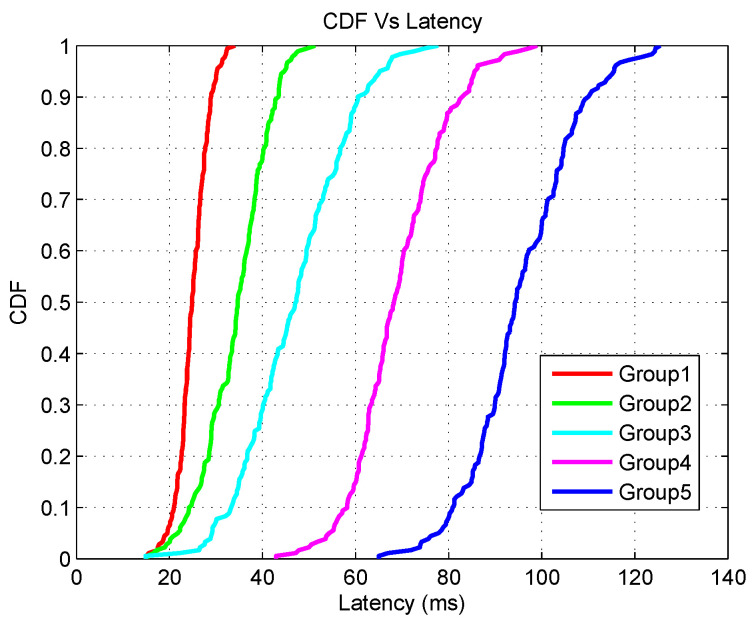
CDF Vs Latency.

An analysis is carried out to understand the performance of various groups of applications in terms of latency. Figure 9 shows that at the 90th percentile point, the latency of the application group1 is the lowest whereas the latency of the group5 application is the highest. The group1 application experiences a latency of 29.34 ms whereas group5 experiences a latency of 110.5 ms at the 90th percentile point. This demonstrates that any packet of group1 has a probability of 90% of reaching the destination gateway within 29.34 ms and in 10% of the time, the packet may not reach the destination successfully or it might take higher than 29.34 ms. Similarly, for the data packet of application group5, any packet has a probability of 90% of being delivered successfully within a 110.5 ms time period while 10% of the time the packet may not be delivered or it might take more than 110.5 ms. The result is obvious due to the fact that more time is required for the controller to resolve the *packet_in* request as the number of applications increases.

In our previous work [32], we have chosen an arbitrary number of controllers to simulate the performance study of software-defined application-specific traffic management for WBANs, whereas in this paper a mathematical model has been developed and then, based on the analytical output to find the optimal number of controllers, the simulation result is provided.

## 6. Conclusions and Future Work

In this study, an optimization model for the design of the control plane for SDWBAN framework has been developed to facilitate healthcare applications. The derived mathematical model leads to a relationship between the number of controllers, SDESWs and WBAN sensors. Ultimately, from the analytical output, the optimal number of controllers turns out to be 5 for the initial implementation of the SDWBAN framework for a delay boundary of 110 ms. The analytical output is then validated by varying the number of controllers while other physical resources in the simulation remain similar to the initial one. The simulation output indicates the optimal point is 6 for both the average PDR and latency analysis of application groups 1 to 5. However, based on the simulation output, the average PDR and latency observed in the range of 4 to 6 controllers are quite acceptable. The outcome of the proposed mathematical model supports the control plane design of the SDWBAN framework.

In future work, it would be interesting to observe the PDR and latency by varying the number of SDESWs while keeping the number of controllers in the range of 4 to 6. This will enable us to visualize the effect of flow requests originating from a various number of SDESWs. The service rate of the controller could be varied in the optimization model since the higher the service rate, the better the flow resolve rate. Therefore, fewer number of controllers would be able to support a higher number of SDESWs. In addition, the optimization model does not consider the delay incurred in getting channel access. In the future, the back-off time or time to get access in the channel can be included as well.

## Figures and Tables

**Table 1:** List of Abbreviations.

Abbreviation	Elaboration
WBAN	Wireless Body Area Network
SDN	Software-Defined Networking
SDWBAN	SDN-based WBAN
PDR	Packet Delivery Ratio
SDESW	SDN-enabled Switch
QoS	Quality of Service

**Table 2:** Notations and Meaning.

Notations	Meaning
$T_{FR}$	Flow Request Delay
$T_{q}$	Queuing Delay
$T_{proc}$	Processing Delay
$T_{prop1}$	Propagation Delay—SDESW to Controller
$T_{prop2}$	Propagation Delay—Controller to SDESW
$T_{RD}$	Relaying Delay

**Table 3:** Numerical Co-efficient and Root.

Flow Resolution Time (*T* ms)	Numerical Coefficient (*c*)	Root ($\sqrt{\theta }$)
20	−0.0099	(0.4107, −2.4117)
30	−0.0199	(0.7292, −2.7302)
40	−0.0299	(0.9976, −2.9986)
50	−0.0399	(1.2340, −3.2349)
60	−0.0499	(1.4476, −3.4486)
70	−0.0599	(1.6441, −3.6451)
80	−0.0699	(1.8269, −3.8279)
90	−0.0799	(1.9986, −3.9996)
100	−0.0899	(2.1610, −4.1620)
110	−0.0999	(2.3155, −4.3165)
120	−0.1099	(2.4631, −4.4640)
130	−0.1199	(2.6046, −4.6056)
140	−0.1299	(2.7408, −4.7417)
150	−0.1399	(2.8722, −4.8731)
160	−0.1499	(2.9993, −5.002)

**Table 4:** Optimal Controllers.

Number of Body Sensors (S)	Optimal Controllers
100	5
200	10
300	14
400	19
500	24

**Table 5:** List of Parameters.

Parameters	Values
Flow Request Delay, $T_{FR}$	100 μs [34]
Free space propagation speed, *C*	$3\times 10^{8}$ m/s
Average length of a hop from SDESW to Controller, $d_{avg}$	32.30 m
Propagation Delay in one hop, $T_{hop}$	$T_{hop}=d_{avg}/C$
Storing and Forwarding delay, $T_{SF}$	20 ms
Packet Arrival Rate, λ	50 pkt/s [23]
Service Rate, α	100 pkt/s
Maximum Queue Size, *K*	15

**Table 6:** Group of Applications.

Groups	Number of Applications
Group 1	1
Group 2	5
Group 3	10
Group 4	15
Group 5	20

**Table 7:** Simulation Parameters (adapted from our previous work Hasan et al. [32]).

Parameter	Value(s)
Simulation Area	75 × 75 m${}^{2}$
Radio range (BS, SDESW, Controller)	∼8 m, ∼20 m, ∼20 m
Reference Distance ($d_{0}$)	1 m
Transmission Power (SDESW, BS)	0 dBm, −10 dBm
Data Rate, Modulation Type, Bits Per Symbol, Bandwidth	250 Kbps, PSK, 4, 20 MHz
Number of BS, Gateway	100, 4
Noise Bandwidth, Noise Floor, Sensitivity	194 MHz, −100 dBm, −95 dBm
BS density	4 nodes/225 m${}^{2}$
Free Space Path Loss exponent	2.4
Total SDESW	25 (1 node per sector)
Initial Average Path Loss ($PL(d_{0})$)	55 dB
Total Controller	4 (2 × 2 grid)
Gaussian Zero-Mean Random Variable (X)	4.0
Number of Clusters	25

## Appendix A

**Proof** **of** **Lemma.** Let us assume that the total number of hop counts in the building grid is *N*. The number of hops from the SDESWs to reach the controller located at the center are $n_{1},n_{2},n_{3},...,n_{\theta }$. Now, considering the hop counts row-wise from the SDESWs to the controller, the total number of hops is:

(A1) $$\begin{array}{cc} N & =\left(n_{1}+n_{2}+n_{3}+---+n_{\sqrt{\theta }}\right)+\left(n_{\sqrt{\theta }+1}+n_{\sqrt{\theta }+2}+n_{\sqrt{\theta }+3}+---+n_{2\sqrt{\theta }}\right)\\ \; & +\left(n_{2\sqrt{\theta }+1}+n_{2\sqrt{\theta }+2}+n_{2\sqrt{\theta }+3}+---+n_{3\sqrt{\theta }}\right)+---+(n_{\left(\sqrt{\theta }-1\right)\left(\sqrt{\theta }+1\right)}\\ \; & +n_{\left(\sqrt{\theta }-1\right)\left(\sqrt{\theta }+2\right)}+n_{\left(\sqrt{\theta }-1\right)\left(\sqrt{\theta }+3\right)}+---+n_{\theta }) \end{array}$$

The hops are counted horizontally until they reach the intersection of the controller’s column. Assuming the controller is located at the center of the grid and hops are counted in both X-Y directions therefore,

(A2) $$\begin{array}{cc} N & =\left\{\left(\sqrt{\theta }-1\right)+\left(\sqrt{\theta }-2\right)+---+(\sqrt{\theta }-\left(\frac{\sqrt{\theta }+1}{2}\right)+---+\left(\sqrt{\theta }-2\right)+\left(\sqrt{\theta }-1\right)\right\}\\ \; & +\left\{\left(\sqrt{\theta }-2\right)+\left(\sqrt{\theta }-3\right)+---+(\sqrt{\theta }-\left(\frac{\sqrt{\theta }+1}{2}\right)-1+---+\left(\sqrt{\theta }-3\right)+\left(\sqrt{\theta }-2\right)\right\}\\ \; & +\left\{\left(\sqrt{\theta }-3\right)+\left(\sqrt{\theta }-4\right)+---+(\sqrt{\theta }-\left(\frac{\sqrt{\theta }+1}{2}\right)-2+---+\left(\sqrt{\theta }-4\right)+\left(\sqrt{\theta }-3\right)\right\}\\ \; & +---+\left\{\left(\sqrt{\theta }-1\right)+\left(\sqrt{\theta }-2\right)+---+(\sqrt{\theta }-\left(\frac{\sqrt{\theta }+1}{2}\right)+---+\left(\sqrt{\theta }-2\right)+\left(\sqrt{\theta }-1\right)\right\} \end{array}$$

(A3) $$\begin{array}{cc} N & =\left\{\theta -\frac{{\left(\sqrt{\theta }+1\right)}^{2}}{4}\right\}+\left\{\theta -\frac{{\left(\sqrt{\theta }+1\right)}^{2}}{4}-\sqrt{\theta }\right\}+\left\{\theta -\frac{{\left(\sqrt{\theta }+1\right)}^{2}}{4}-2\sqrt{\theta }\right\}\\ \; & +---+\left\{\theta -\frac{{\left(\sqrt{\theta }+1\right)}^{2}}{4}\right\} \end{array}$$

Finally, the average number of hops *n* from a SDESW to the controller is,

(A4) $$n=\frac{N}{\theta }≅\frac{\sqrt{\theta }}{2}$$

□

## References

[1] Islam S.R., Kwak D., Kabir M.H., Hossain M., Kwak K.S. (2015). The internet of things for health care: A comprehensive survey. IEEE Access.

[2] Pramanik P.K.D., Solanki A., Debnath A., Nayyar A., El-Sappagh S., Kwak K.S. (2020). Advancing Modern Healthcare With Nanotechnology, Nanobiosensors, and Internet of Nano Things: Taxonomies, Applications, Architecture, and Challenges. IEEE Access.

[3] Darwish A., Hassanien A.E. (2011). Wearable and implantable wireless sensor network solutions for healthcare monitoring. Sensors.

[4] Bouazizi A., Zaibi G., Samet M., Kachouri A. Wireless body area network for e-health applications: Overview. Proceedings of the 2017 International Conference on Smart, Monitored and Controlled Cities (SM2C).

[5] Hasan K., Biswas K., Ahmed K., Nafi N.S., Islam M.S. (2019). A comprehensive review of wireless body area network. J. Netw. Comput. Appl..

[6] Kreutz D., Ramos F.M.V., Veríssimo P.J.E., Rothenberg C.E., Azodolmolky S., Uhlig S. (2015). Software-Defined Networking: A Comprehensive Survey. Proc. IEEE.

[7] Ahmad I., Namal S., Ylianttila M., Gurtov A.V. (2015). Security in Software Defined Networks: A Survey. IEEE Commun. Surv. Tutor..

[8] Cox J.H., Chung J., Donovan S., Ivey J., Clark R.J., Riley G., Owen H.L. (2017). Advancing software-defined networks: A survey. IEEE Access.

[9] Nkenyereye L., Nkenyereye L., Islam S.M.R., Choi Y., Bilal M., Jang J. (2019). Software-Defined Network-Based Vehicular Networks: A Position Paper on Their Modeling and Implementation. Sensors.

[10] Hasan K., Wu X., Biswas K., Ahmed K. A Novel Framework for Software Defined Wireless Body Area Network. Proceedings of the International Conference on Intelligent Systems, Modelling and Simulation.

[11] Bari M.F., Roy A.R., Chowdhury S.R., Zhang Q., Zhani M.F., Ahmed R., Boutaba R. Dynamic Controller Provisioning in Software Defined Networks. Proceedings of the International Conference on Network and Service Management.

[12] Levin D., Wundsam A., Heller B., Handigol N., Feldmann A. (2012). Logically Centralized?: State Distribution Trade-offs in Software Defined Networks. Workshop on Hot Topics in Software Defined Networks.

[13] Heller B., Sherwood R., McKeown N. (2012). The Controller Placement Problem. Workshop on Hot Topics in Software Defined Networks.

[14] Hu T., Guo Z., Yi P., Baker T., Lan J. (2018). Multi-controller Based Software-Defined Networking: A Survey. IEEE Access.

[15] Singh A.K., Srivastava S. (2018). A survey and classification of controller placement problem in SDN. Int. J. Netw. Manag..

[16] Xie J., Guo D., Hu Z., Qu T., Lv P. (2015). Control plane of software defined networks: A survey. Comput. Commun..

[17] Sallahi A., St-Hilaire M. (2015). Optimal Model for the Controller Placement Problem in Software Defined Networks. IEEE Commun. Lett..

[18] Hock D., Hartmann M., Gebert S., Jarschel M., Zinner T., Tran-Gia P. Pareto-optimal resilient controller placement in SDN-based core networks. Proceedings of the International Teletraffic Congress.

[19] Yao G., Bi J., Li Y., Guo L. (2014). On the Capacitated Controller Placement Problem in Software Defined Networks. IEEE Commun. Lett..

[20] Jiménez Y., Cervelló-Pastor C., García A.J. On the controller placement for designing a distributed SDN control layer. Proceedings of the 2014 IFIP Networking Conference.

[21] Faragardi H.R., Vahabi M., Fotouhi H., Nolte T., Fahringer T. (2018). An efficient placement of sinks and SDN controller nodes for optimizing the design cost of industrial IoT systems. Softw. Pract. Exp..

[22] Ren W., Sun Y., Luo H., Guizani M. (2019). A Novel Control Plane Optimization Strategy for Important Nodes in SDN-IoT Networks. IEEE Internet Things J..

[23] Liyanage K.S.K., Ma M., Chong P.H.J. (2018). Controller placement optimization in hierarchical distributed software defined vehicular networks. Comput. Netw..

[24] Bagaa M., Taleb T., Laghrissi A., Ksentini A., Flinck H. (2018). Coalitional Game for the Creation of Efficient Virtual Core Network Slices in 5G Mobile Systems. IEEE J. Sel. Areas Commun..

[25] Zhao Z., Wu B. (2017). Scalable SDN architecture with distributed placement of controllers for WAN. Concurr. Comput..

[26] Gorkemli B., Tatlicioglu S., Tekalp A.M., Civanlar S., Lokman E. (2018). Dynamic Control Plane for SDN at Scale. IEEE J. Sel. Areas Commun..

[27] The Cisco Application Policy Infrastructure Controller. https://www.cisco.com/c/en_au/products/cloud-systems-management/application-policy-infrastructure-controller-apic/index.html.

[28] Yao L., Hong P., Zhou W. Evaluating the controller capacity in software defined networking. Proceedings of the 2014 23rd International Conference on Computer Communication and Networks (ICCCN).

[29] Tootoonchian A., Gorbunov S., Ganjali Y., Casado M., Sherwood R. (2012). On Controller Performance in Software-defined Networks. Proceedings of the USENIX Conference on Hot Topics in Management of Internet, Cloud, and Enterprise Networks and Services.

[30] AlGhadhban A., Shihada B. Delay analysis of new-flow setup time in software defined networks. Proceedings of the NOMS 2018—2018 IEEE/IFIP Network Operations and Management Symposium.

[31] Sufiev H., Haddad Y., Barenboim L., Soler J. (2019). Dynamic SDN Controller Load Balancing. Future Internet.

[32] Hasan K., Ahmed K., Biswas K., Islam M.S., Sianaki O.A. (2020). Software-defined application-specific traffic management for wireless body area networks. Future Gener. Comput. Syst..

[33] Nafi N.S., Ahmed K., Gregory M.A., Datta M. (2018). Software defined neighborhood area network for smart grid applications. Future Gener. Comput. Syst..

[34] Holmberg K., Adgar A., Arnaiz A., Jantunen E., Mascolo J., Mekid S. (2010). E-Maintenance.

[35] Zhu L., Karim M.M., Sharif K., Li F., Du X., Guizani M. (2019). SDN Controllers: Benchmarking & Performance Evaluation. arXiv.

[36] Boulis A. (2011). Castalia 3.2, User’S Manual.

